# Cholesterol Gallstones and Long-Term Use of Statins: Is Gut Microbiota Dysbiosis Bridging over Uncertainties?

**DOI:** 10.3390/diagnostics14121234

**Published:** 2024-06-12

**Authors:** Doina Georgescu, Daniel-Florin Lighezan, Ioana Ionita, Nicoleta Hadaruga, Roxana Buzas, Ciprian-Ilie Rosca, Mihai Ionita, Ioana Suceava, Diana-Alexandra Mitu, Oana-Elena Ancusa

**Affiliations:** 1 Department V of Internal Medicine I, “V Babeș” University of Medicine and Pharmacy, 300041 Timisoara, Romania; doina.georgescu@umft.ro (D.G.); dlighezan@umft.ro (D.-F.L.); buzas.dana@umft.ro (R.B.); rosca.ciprian@umft.ro (C.-I.R.); ionita.mihai@umft.ro (M.I.); suceava.ioana@umft.ro (I.S.); diana-alexandra.mitu@umft.ro (D.-A.M.); ancusa.oana@umft.ro (O.-E.A.); 2 Department of Food Science, University of Life Sciences “King Michael I”, 300041 Timisoara, Romania; nicoletahadaruga@usvt.ro

**Keywords:** gallstones, gut microbiota imbalance, cholesterol-lowering agents

## Abstract

A total of 300 research participants—200 consecutive patients diagnosed with dyslipidemia (100 statin (+), treated for at least five years, and 100 statin (−)) and 100 healthy controls—were included in this observational study. The aim of the study was to deliver insights into the relationship between the long-term use of statins for dyslipidemia and gallstone disease (GSD), as well as insights into the background particularities of the gut microbiota. All study participants underwent clinical examination, laboratory workups, stool microbiology/stool 16S r RNA, next-generation sequencing, and abdominal ultrasound/CT exams. Results: The research participants presented with similarities related to age, gender, and location. Patients displayed comparable heredity for GSs, metabolic issues, and related co-morbidities. Gut dysbiosis (DB) was present in 54% of the statin (−) patients vs. 35% of the statin (+) patients (*p* = 0.0070). GSs were present in 14% of patients in the statin (−) group vs. 5% of patients in the statin (+) group (*p* = 0.0304). Severe dysbiosis, with a significant reduction in biodiversity, an increase in LPS (+) bacteria, and a notable decrease in mucin-degrading bacteria, mucosa-protective bacteria, and butyrate-producing bacteria were observed in the statin (−) group. Strong positive correlations between GSD and diabetes/impaired glucose tolerance (r = 0.3368, *p* = 0.0006), obesity (r = 0.3923, *p* < 0.0001), nonalcoholic fatty liver disease (r = 0.3219, *p* = 0.0011), and DB (r = 0.7343, *p* < 0.0001), as well as significant negative correlations between GSD and alcohol use (r = −0.2305, *p* = 0.0211), were observed. The multiple regression equation demonstrated that only DB (95% CI: 0.3163 to 0.5670; *p* < 0.0001) and obesity (95% CI: 0.01431 to 0.2578; *p* = 0.0289) were independent risk factors predicting GSD in the group of patients treated with statins. Conclusion: The long-term use of statins in dyslipidemic patients was associated with a low risk of developing GSs. The gut microbiota associated with a long-term use of statins in dyslipidemic patients was characterized by a low risk of developing an imbalance of various functional bacteria and alterations in the metabolic microbiota. DB and obesity were found to be independent risk factors predicting GSD in statin (+) patients.

## 1. Introduction

Gallstones (GSs) represent the presence of various types of calculi within the biliary tree, most frequently located at the gallbladder (GB) level or in the common bile duct. They have several sizes, shapes, and consistencies, depending on their age and chemical composition [1]. GSs result from a process of precipitation of constituents from the bile when the balance between various compounds breaks, causing the transformation of soluble chemical compounds into a solid state as microcrystals, which further grow into visible shapes as stones. Several factors could trigger lithogenic bile, some of which are related to the hepatocyte’s ability to secrete excess chemical substances, eventually resulting in a modified bile, while others intervene at the level of GB motility with a decrease in GB emptying, favoring an overconcentration or supersaturation (biliary sludge) of the bile’s compounds, as well as impaired washout of various types of microcrystals that could eventually develop in the process [2,3,4]. GSs are currently categorized as cholesterol stones, containing more than 70% cholesterol; pigment stones, containing more than 70% bilirubin (subdivided into black stones caused by calcium bilirubinate and brown stones caused by calcium bilirubinate, tribasic phosphate, and calcium fatty acids); and mixed stones, containing different proportions of cholesterol and bilirubin, as well as different chemical substances such as calcium carbonate, calcium phosphate, and calcium palmitate [5,6]. 

The relation between the pathogenesis, morphology, and chemical composition of GSs has been highlighted by many authors, and various GS classification systems have been proposed. From the historical GS classification based on stasis or infection, the concept evolved to metabolic stones, based on either cholesterol or bilirubinate. According to analyses based on their macroscopic appearance, associating the gross morphology with the chemical composition, GS subtypes are cholesterol GSs, pigment GSs, and mixed stones [7]. The exact composition of a GS is determined by means of X-ray diffraction, as well as physical or chemical analytic qualitative and quantitative methods [8]. GSs, especially cholesterol ones, are highly prevalent in Western countries. Their incidence increases with age and is associated with the female gender and various medical conditions and metabolic disorders [9]. Local factors, related to the GB, may intervene in the development of cholesterol-rich GSs, particularly a motility disfunction with impaired evacuation and build-up of intraluminal mucin, chronic surface epithelial injury resulting in inflammatory reactions, and quantitative and qualitative modifications of the bile, with cholesterol enrichment and precipitation of crystals. Systemic factors related to heredity with gene polymorphism may also be involved, as may many epigenetic factors, cholesterol metabolism perturbations, and various hormonal factors, especially insulin resistance. Except for heredity, most of these factors can be addressed [10]. Over the past decades, there have been tremendous changes in the understanding of the pathogenesis of gallstone disease (GSD). One of the cornerstones of this new approach is represented by the influence of the gut microbiota’s dysbiosis. Many recent research results have highlighted the association between GSD and the imbalance of the gut microbiome, with important changes in the diversity and abundance of the various phyla. By influencing some metabolic pathways of the biliary acids (BAs), microbiota dysbiosis may intervene in the etiopathogenesis of GSD as a lithogenic risk cofactor. In contrast, the implication of the gut microbiome in the regulation of the enterohepatic bile’s acid recycling may interfere with the intestinal cholesterol absorption, promoting GSD [11,12]. Recent studies have reported that patients treated with statins exhibit a lower prevalence of gut dysbiosis, as well as a modification of the microbiota’s composition [13,14]. Statins are commonly used as potent cholesterol-lowering agents, acting as temporary inhibitors of 3-hydroxy-3-metylglutaryl-CoA reductase, while also having multiple pleiotropic effects that are characterized by improving the endothelial function, lowering oxidative stress, and having immunomodulatory, anti-inflammatory, and antithrombotic effects. These beneficial aspects, especially the effect on the cholesterol metabolism, may be closely related to some particularities of the gut microbiota. Beyond these aspects, a bidirectional relationship seems to exist between statins and the intestinal microbiome. Thus, according to some studies, treatment with atorvastatin in patients with hypercholesterolemia may influence the gut microbiota, resulting in important modifications that help restore its biodiversity and the balance between several bacteria [15,16]. Long-term statin use seems to prevent not only ischemic heart disease and cardiovascular events but also the development of GSs. The prevention of GSs, and consequently the decreased need for cholecystectomies, might be related to the lowering of secretion and the saturation of the biliary cholesterol with a limitation of cholesterol crystal build-up, as well as to the modifications of the gut microbiota [17]. 

The present observational study raised questions about statins and the association between the intestinal microbiome and GSD and aimed to deliver insights into the relationship between the long-term use of statins for dyslipidemia and GSD and the gut microbiota’s background particularities.

## 2. Materials and Methods

In this study, 746 in-hospital consecutive dyslipidemic patients admitted to the 1st Clinic of Internal Medicine of the Emergency Municipal Clinical Hospital, Timisoara, Romania, between 15 September 2021 and 15 March 2023 were checked for eligibility. Patients were included in this observational study, after signing a written informed consent form, as pairs of one statin (+) and one statin (−) patient, in order to form two evenly study groups, with each group achieving a final number of 100 participants. In addition, 100 healthy controls were enrolled in the study.

**Figure 1:**
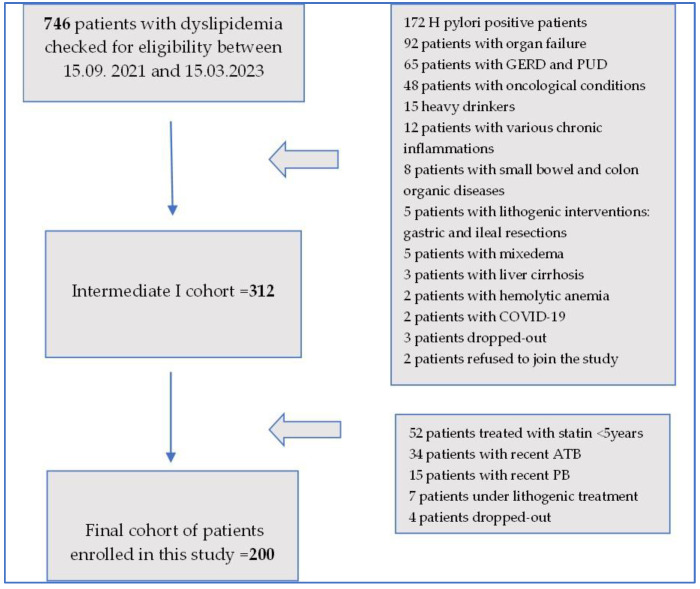
Flowchart of patient inclusion process.

A flowchart of the patient inclusion steps is represented in Figure 1.

As illustrated in Figure 1, out of 746 patients with dyslipidemia, 172 patients had *H. pylori*, 92 patients presented with organ failure, 65 patients had gastroesophageal reflux disease (GERD) and peptic ulcer disease (PUD), 48 patients had oncological conditions, 35 patients were heavy drinkers, 12 patients were found to have various inflammations, 8 patients had small bowel and colon organic conditions, 5 patients had a history of lithogenic interventions with gastric and ileal resections, 5 patients presented with mixedema, 2 patients presented with liver cirrhosis, 2 patients were infected with SARS-CoV-2, and 1 patient had chronic hemolytic anemia. At that stage, 2 patients refused to join the study, and 5 recruited patients dropped out of the study, meaning that the intermediate cohort comprised 312 participants. At that point, out of the 312 patients, 52 patients had been treated with a variety of statins for less than 5 years, 34 had recently been treated with antibiotics (ATBs), 15 were currently under probiotic (PB) treatment, 7 patients were under various lithogenic medications, and another 4 patients dropped out of the study. This meant that by 15 March 2023, a final cohort of 200 patients was achieved. 

Inclusion criteria: Hospitalized patients diagnosed with dyslipidemia who agreed to join the study were included. In order to be a candidate for this study, patients from the statin (+) group were required to have a history of a minimum of 5 years of treatment with statins.

Exclusion criteria: Patients with small bowel and colon organic diseases; severe respiratory, cardiac, liver, or kidney diseases; autoimmune diseases; mixedema; oncological conditions; gastroesophageal reflux and peptic ulcer diseases; *H. pylori* infection; cirrhosis; heavy drinking; chronic hemolytic anemia; or various infections or inflammations such as cholecystitis, pancreatitis, or peritonitis were excluded. In addition, patients receiving fibrates, bile sequestrant, somatostatin, or glucagon-like peptide 2 (GLP-2) receptor agonist treatment; being treated with proton pump inhibitors; receiving corticotherapy or biological therapies; with a recent history of COVID-19 infection; receiving antibiotic or probiotic treatment at enrollment; and who did not agree to join this study were excluded.

### 2.1. Research Participant Approach

Research participants, either patients or controls, went through a thorough clinical examination that included specific data about medications addressing dyslipidemia. Blood pressure measurements were performed in the morning, at rest, in a sitting position. The body mass index was calculated based on height and weight. Routine blood, urine, and stool laboratory workups were performed using standardized methods, accredited by the European Community and Romanian Accreditation Association (RENAR). 

Abdominal ultrasound/CT, gastro-intestinal endoscopy, ECG, and thorax X-ray examinations were also performed on patients who joined the study. Three patients, two from the statin (−) group and one from the statin (+) group, who were diagnosed with GSD had indications for cholecystectomy, which was performed by means of a retrograde laparoscopic approach. After the GB removal, GSs were immediately analyzed in each case with a magnifier glass, using the same protocol: washing with sterile water, careful drying, counting, and description of the gross appearance of their surfaces (shape, size, color). Photos of GSs were taken consecutively. The removed GBs were sent to the Pathology Laboratory for examination (gross description, orientation and paraffin embedding, usual staining, and optic microscopy study). 

The study was carried out in accordance with the Declaration of Helsinki on human rights and was approved by the Ethical Committee of Scientific Research with the University of Medicine and Pharmacy, Timisoara, number 16/24.05.2021.

### 2.2. GSD Diagnostic Methodology 

The imagistic diagnosis of the GSD was mainly based on a Duplex abdominal ultrasound examination or abdominal CT in selected cases [18,19,20]. 

### 2.3. GS Classification and Analysis 

**Figure 2:**
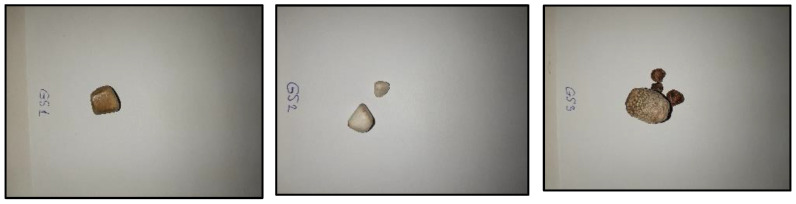
GS samples’ gross appearance: GS1, GS2, GS3a (large gallstone), and GS3b (small gallstones).

**Figure 3:**
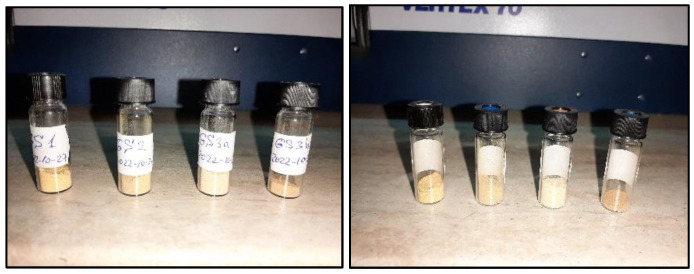
GSs ground in a mortar used for FTIR analysis.

The clinical classification of the GSs was carried out in accordance with the Japanese Society of Gastroenterology [21]. The GS analysis was performed using Fourier transform infrared spectroscopy (FTIR) on a Bruker Vertex 70 device (Bruker Optik GmbH, Ettlingen, Germany) [22,23]. The FTIR had the ability to analyze powdery samples using the single-reflection platinum diamond attenuated total reflectance unit (ATR). The detector (DLaTGS) worked on a 12,000–250 cm^−1^ spectrum range, with a sensibility of D* > 2108 cm·Hz^1/2^·W^−1^. The following FTIR analysis conditions were used: acquisition range 4000–400 cm^−1^, resolution 4 cm^−1^, scans/sample 64, and sample mass 20–50 mg. The GSs used in this study are presented in Figure 2 and Figure 3. They were coded as GS1, GS2, GS3a, and GS3b and were ground in a mortar in order to obtain a homogenous powder for analysis.

### 2.4. Dyslipidemia Diagnostic Methodology 

The dyslipidemia and treatment approach were conducted based on the 2021 ESC Guidelines on cardiovascular disease prevention in clinical practice as follows: blood cholesterol > 200 mg/dL (5.2 mmol/L), triglycerides > 150 mg/dL (1.7 mmol/L), LDL cholesterol > 100 mg/dL (2.58 mmol/L), and/or HDL cholesterol < 40 mg/dL (1.03 mmol/L) [24].

### 2.5. Diabetes Mellitus Diagnostic Methodology

Diabetes mellitus (DM) and impaired glucose tolerance (IGT) were diagnosed according to the American Diabetes Association’s criteria [25]. 

### 2.6. Microbiological and Sequencing Assessment of Stools 

The stool samples were collected using sterile standardized containers and frozen at −20 °C. Different types of stool species, such as aerobe, anaerobe, or microaerophiles, were initially identified by means of the matrix-assisted laser desorption ionization–time of the flight–mass spectrometry (MALDI-TOF-MS) method and expressed as colony-formatting units (CFUs)/gram stool. Additional comments were provided by the laboratory as follows: physiological condition (normobiosis), borderline situations (±), or gut dysbiosis, represented either by an increase (+) or reduction (−) in diverse microorganisms. These alterations of the gut microbiota were semi-quantitatively expressed as follows: (+ or −) = mild dysbiosis, (++ or −−) = moderate dysbiosis, and (+++ or −−−) = severe dysbiosis. In order to input the dysbiosis into the statistical analysis as not only a categorical variable but also as a quantitative variable, a numerical equivalence of the laboratory-provided data was performed. Accordingly, the numerical equivalence of the severity of the dysbiosis was carried out in the following way: mild—1 point, moderate—2 points, and severe—3 points [26]. 

The stool samples from the patients with DB were further processed by means of the 16S r RNA next-generation sequencing method. The enterotypes of the gut microbiota were set as follows: enterotype I *Bacteroides* spp. predominant, enterotype II *Prevotella* spp. predominant, enterotype III *Ruminococcus* spp. predominant, and unclassified enterotypes. The H index of the alpha biodiversity, several bioindicators, and the functional and metabolic bacteria of the gut microbiome were assessed [27].

A statistical analysis was performed using Graph Pad Prism ver.10.1.0 (316) software (Graph Pad Software, Inc., La Jolla, CA, USA). Quantitative variables were expressed as mean values (MVs) ± standard deviation (SD). The chi-squared test is a statistical tool, which was used to check if two categorical variables, expressed as percentages, were related or independent. The unpaired *t*-test was calculated, and *p* ≤ 0.05 was considered statistically significant, with a confidence interval CI = 95%. The frequency distribution of the data was analyzed, and representative histograms were drawn. The nonparametric Pearson’s correlation test was performed with the calculation of the “r^2^” coefficient in order to assess the magnitude and direction of possible correlations. The relationship between variables was expressed as an equation of linear regression, and graphs were drawn consecutively. Multivariate analyses were used consecutively to estimate the relationship between selected independent variables, represented by various clinical data, and one dependent variable, represented by GSD, and to rule out possible confounders. The OPUS ver. 7.2 software from the Bruker Vertex 70 equipment manufacturer was used for the acquisition and handling of the FTIR data. All FTIR determinations were performed in triplicate (coded as “a”, “b”, and “c”), both for the gallstone samples and the cholesterol reference compound. Hierarchical cluster analysis (HCA) and statistical analysis were performed using Microsoft Excel 2016 from the Microsoft Office Professional Plus 2016 package. FTIR wavenumbers and intensities were evaluated as the mean (±standard deviation) of triplicate determinations. Both the specific FTIR data from the triplicates and the mean values were used for the hierarchical cluster analysis (HCA) using the statistical package. The “tree clustering” method with a single-linkage amalgamation rule and measured using non-standardized Euclidean distances was applied for the HCA.

## 3. Results

This is an observational study that analyzed 300 research participants: 200 consecutive patients with dyslipidemia and 100 healthy controls.

As seen in Table 1, our analysis of the demographic chart revealed that the research participants displayed no significant differences related to age, gender, residency, or occupational activities.

Biological parameters, such as the cell blood count, C-reactive protein, alanine aminotransferase, conjugated bilirubin, pancreatic enzymes, fast plasma glucose, creatinine, low-density lipoproteins (LDLs), triglycerides, and gut dysbiosis, were analyzed and are presented in Table 2. These laboratory workups were compared between the two groups of patients, statin (+) vs. statin (−), and between patients and controls.

As seen in Table 2, significant differences were observed for LDL cholesterol, triglycerides, and gut dysbiosis, with patients from the statin (+) group displaying lower levels. Fast plasma glucose was significantly higher in patients from the statin (+) group. 

Various clinical data on patients with dyslipidemia, either in the statin (+) or statin (−) group, are presented in Table 3. 

As depicted in Table 3, no significant differences were recorded concerning admission, family history of GSD, alcohol drinking or cigarette smoking, female postmenopausal estrogen replacement therapy, gastro-intestinal conditions, DM or IGT, metformin treatment, hypertension and other cardiovascular diseases, as well as in-hospital outcome. The statin (+) group displayed statistically significant differences related to a longer duration of dyslipidemia and a lower incidence of GSD.

Table 4 displays the modifications to the stool microbiome in both groups, statin (+) and statin (−), in terms of bioindicators and functional bacteria, as well as in terms of alterations to the bacterial metabolism. 

As seen in Table 4, the dysbiosis severity was significantly lower in the statin (+) group. Our study of the enterotypes demonstrated that significant differences were present only for enterotype 1 (*Bacteroides* spp. predominant), with patients from the statin (−) group exhibiting a higher incidence of this enterotype. The bioindicators of the gut microbiota represented by the alpha biodiversity index demonstrated that patients from the statin-free group exhibited a significantly lower index of biodiversity. The functional bacteria, such as mucosa-protective and mucin-degrading microbiota, displayed significant differences, characterized by a decreased range in the statin-free group, unlike LPS (+) bacteria, which were significantly higher in the statin-free group. Regarding the bacterial metabolism, the patients from the statin-free group frequently showed a decrease in the butyrate-producing microbiota.

**Figure 4:**
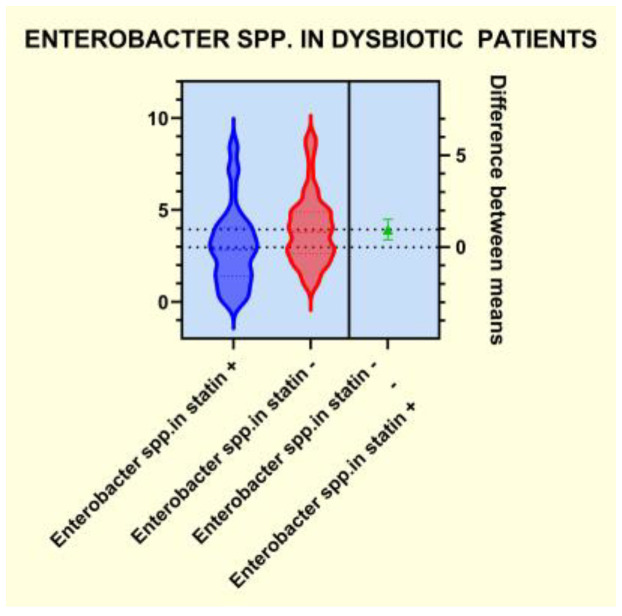
Distribution of *Enterobacter spp*. in dysbiotic patients.

**Figure 5:**
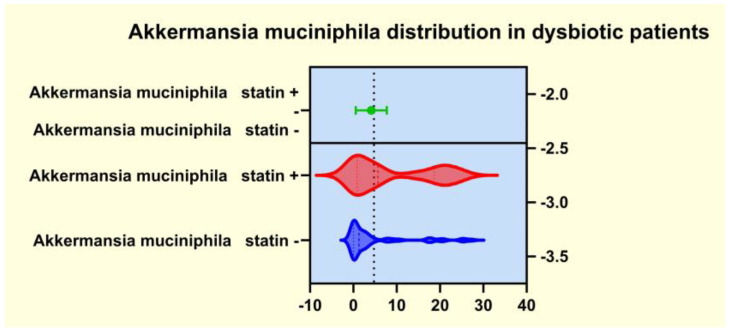
Distribution of *Akkermansia muciniphila* in dysbiotic patients.

**Figure 6:**
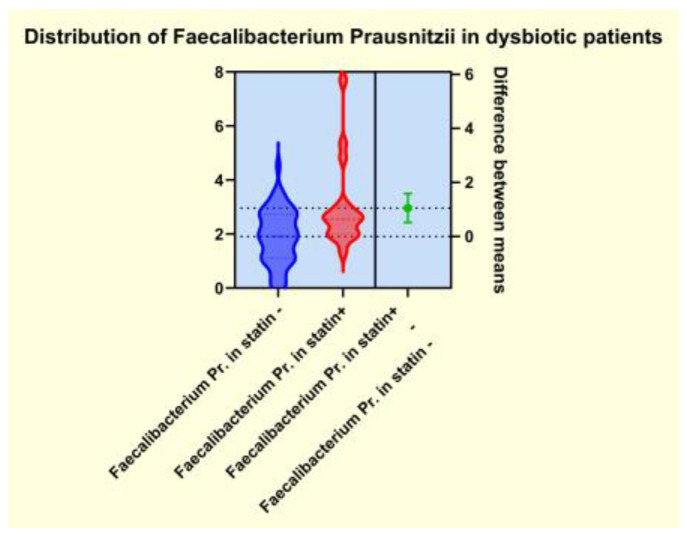
Distribution of *Faecalibacterium Prausnitzii* in dysbiotic patients.

**Figure 7:**
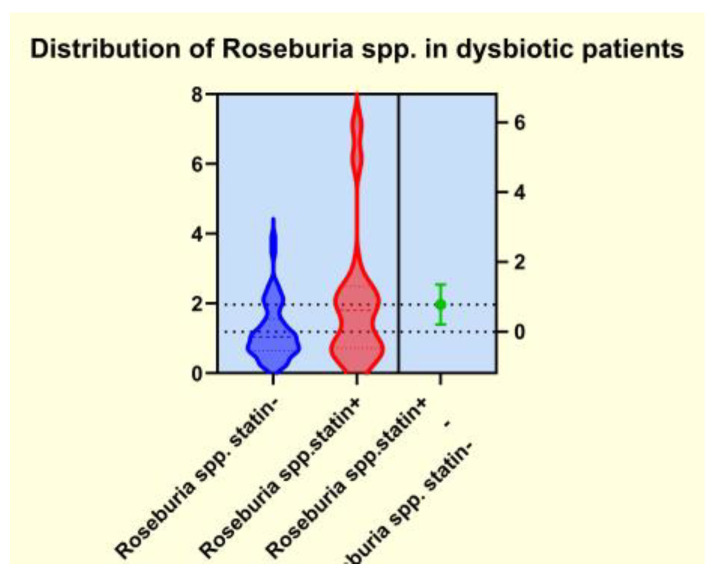
Distribution of *Roseburia spp*. in dysbiotic patients.

As presented in Figure 4, Figure 5, Figure 6 and Figure 7, the analyses of several bacterial strains revealed significant differences, with higher or lower ranges of particular species, as follows: *Enterobacter* spp. were found to be significantly elevated in the statin-free group (3.941 vs. 2.872 *p* = 0.0007; Figure 4), and other species were found to be significantly decreased in the statin-free group, such as *Akkermansia muciniphila* (4.751 vs. 8.905, *p* = 0.0241; Figure 5), *Faecalibacterium Prausnitzii* (1.911 vs. 2.966, *p* = 0.0002; Figure 6), and *Roseburia* spp. (1.193 vs. 1.972, *p* = 0.0084; Figure 7).

The clinical aspects related to patients with GSs, both those who were treated with and without statins, are presented in Table 5.

As seen in Table 5, statistically significant differences were only noted in relation to the GSs’ size. The patients from the statin (+) group exhibited GSs with smaller sizes, while the patients from the statin (−) group had GSs of larger sizes. The other analyzed aspects showed comparable data. 

It is worth noting that the GSs from both groups proved to be cholesterol-rich stones, meaning that the amount of cholesterol was above 70% of their dry weight. The FTIR analysis is presented in Table 6.

**Figure 8:**
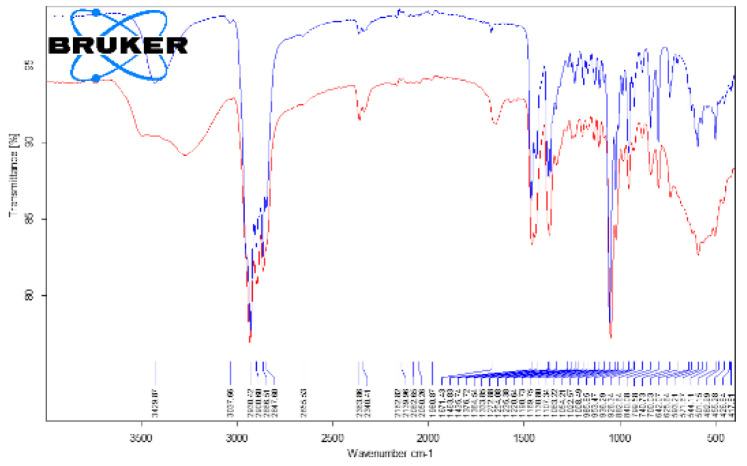
Superimposed FTIR spectra of cholesterol (reference compound (blue)) and gallstone “*GS1*” (red), corresponding to whole range of 4000–400 cm^−1^.

**Figure 9:**
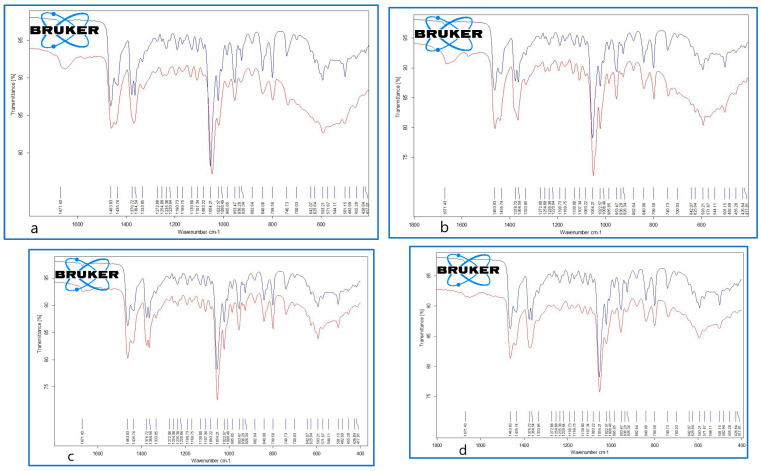
Superimposed FTIR spectra of cholesterol (reference compound (blue)) and gallstone samples, *GS1* (**a**), upper left; *GS2* (**b**), upper right; *GS3a* (**c**), bottom left; and *GS3b* (**d**), bottom right (red), corresponding to the representative range of 1800–400 cm^−1^.

The representative FTIR spectra for the cholesterol (reference compound, analytical grade) and the studied gallstones *GS1*, *GS2*, *GS3a*, and *GS3b* are presented in Figure 8 and Figure 9. We used a reference compound and the gallstone samples (GS1, GS2, GS3a, and GS3b; see Figure 3 for codes); * indicates specific bands of bilirubin.

The FTIR data of the GSs revealed the presence of both cholesterol and bilirubin compounds in the composition, with a higher content of cholesterol in the examined samples. Other FTIR aspects are available as supplementary files, such as the following: Figure S1a. Superimposed FTIR spectra for cholesterol (triplicate samples), Figure S1b. Superimposed FTIR spectra for cholesterol as reference compound (blue) and gallstone GS1(red), Figure S1c. Superimposed FTIR spectra for cholesterol as reference compound (blue) and gallstone GS2(red), Figure S1d. Superimposed FTIR spectra for cholesterol as reference compound (blue) and gallstone GS3a. Figure S1e. Superimposed FTIR spectra for cholesterol as reference compound (blue) and gallstone GS3bl (red), corresponding to the full range of 4000–400 cm^−1^.

**Figure 10:**
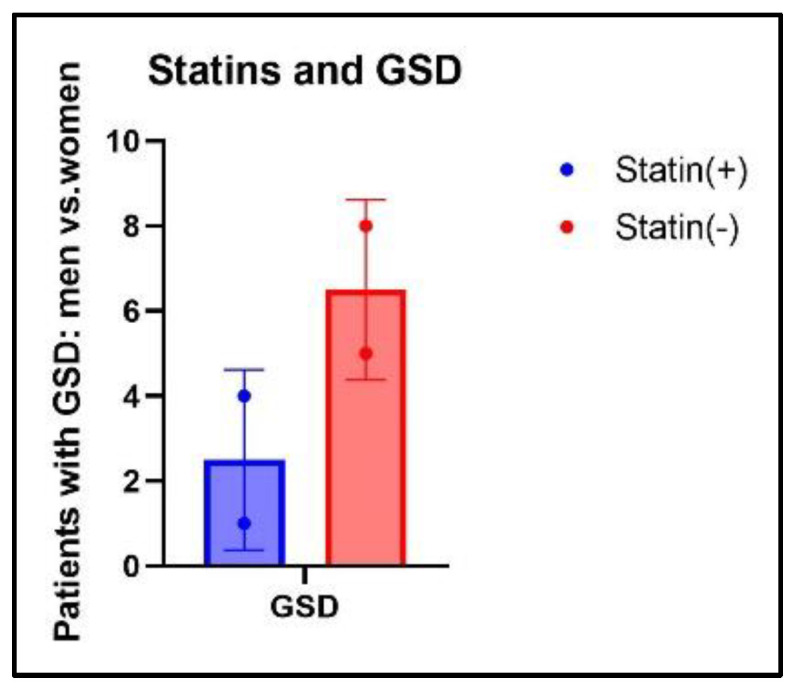
Distribution of GSs related to gender ratio and treatment.

As illustrated in Figure 10, the female gender was prevalent in both groups with GSD, accounting for 64.28% of the statin (−) group and 80% of the statin (+) group. 

**Figure 11:**
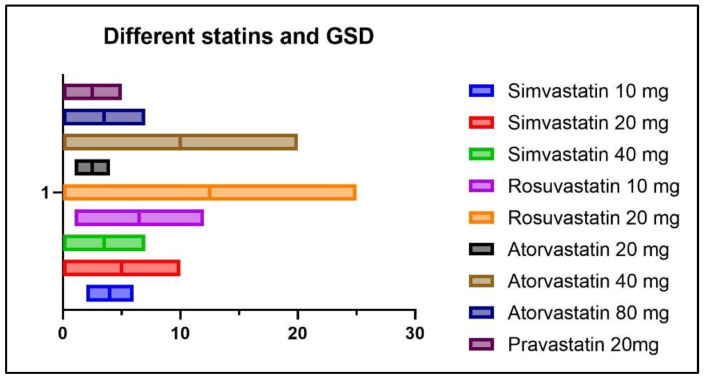
Distribution of GSD according to statin formulation and dosage.

The distribution of GSD in relation to the different statin formulations and dosages in statin (+) patients is illustrated in Figure 11.

As seen in Figure 11, out of a total of five patients with GSD from the statin (+) group, two patients were treated with Simvastatin 10 mg/day, two patients were treated with Rosuvastatin 10mg/day, and one patient was treated with Atorvastatin 20 mg/day. 

**Figure 12:**
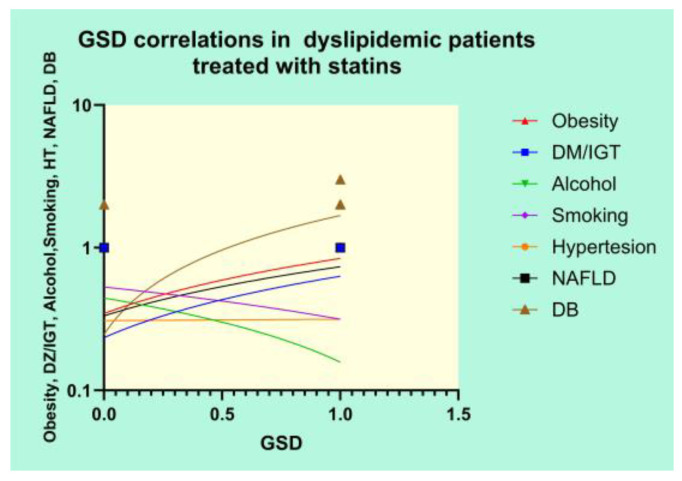
Correlational graph of GSD and clinical variables.

The correlations between GSD in patients treated with statins and several clinical variables are depicted in Figure 12.

The Pearson’s results of the correlation analysis revealed strong positive correlations between GSD and DM/IGT (r = 0.3368, *p* = 0.0006), obesity (r = 0.3923, *p* < 0.0001), NAFLD (r = 0.3219, *p* = 0.0011), and DB (r = 0.7343, *p* < 0.0001) and a significant negative correlation between GSD and alcohol use (r = −0.2305, *p* = 0.0211). 

In order to determine which variables are independent in the development of GSD, we ran a multivariate analysis. The dependent set variable was GSD in patients treated with statins. The results are represented in Table 7. 

As seen in Table 7, the same variables that were analyzed using Pearson’s correlation test were also included in the model for the multiple regression analysis. The results of the multiple regression equation emphasized that only DB (95% CI: 0.3163 to 0.5670; *p* < 0.0001) and obesity (95% CI: 0.01431 to 0.2578; *p* = 0.0289) were independent risk factors predicting GSD in the group of patients treated with statins.

## 4. Discussion

GSD with cholesterol-rich stones is highly associated with many known risk factors, such as heredity, age and gender, environmental factors, lifestyle, dietary habits, and various metabolic conditions, as we also observed in the present study. At least in theory, every pathogenic factor that is associated with the development of cholesterol-rich GSs may at some point be addressed by means of therapeutical interventions in order to treat or even prevent GSD [28,29,30,31]. The risk factors analyzed in this study emphasized that patients treated with statins showed strong positive correlations between GSD and DZ/IGT, obesity, NAFLD, and DB and a significant negative correlation between GSD and a low alcohol intake. Hypertension and smoking cigarettes did not show any significant correlations with GSD. However, the multivariate analysis demonstrated that not all the variables that strongly correlated with GSD were independent risk factors, meaning that only DB and obesity were independent risk factors predicting GSD in the group of patients treated with statins. These two risk factors that were emphasized in the present study might be addressed in order to influence the development of GSD.

Many patients with GSD have an insidious form of the disease and remain asymptomatic throughout their life. Our results have shown that 42.10% of the patients with GSD were asymptomatic, being randomly diagnosed when an abdominal ultrasound was performed. The rest of the patients were symptomatic, with 31.57 complaining of dyspepsia and only 26.31% presenting with clinical signs such as jaundice and positivity of Murphy’s maneuver. Interestingly, patients from the group treated with statins exhibited smaller-sized GSs and tended to present with latent or mild forms of GSD.

The GSs from patients from Western countries are mainly cholesterol-rich, originating from the cholesterol-supersaturated bile. Given that the statins act as inhibitors of the hepatic cholesterol synthesis, they may theoretically decrease the risk of GSD. Meta-analyses and case–control studies have revealed that patients receiving statins are at a lower risk of developing GSD [32]. In contrast, according to some studies, the effect of statins on GSD remains controversial and a subject of debate [33,34]. Other studies, such as a retrospective cohort study of 6342 patients who were either treated with statins or not, revealed that statins did not influence GSD, neither by favoring its apparition nor by protecting against it [35]. However, a recent study of a large cohort of Asian patients who were treated long-term with any statin formulation or only with lipophilic statins demonstrated lower odds of the incidence of GSD [36]. Recently, a meta-analysis studied several aspects of the relationship between statin use and the incidence of GSD, including the duration of the treatment. The results of this study emphasized that short-term use was not associated with a decreased incidence of GSD; however, patients who used statins for more than five years had a significantly lower risk of GSD compared to nonusers [37]. The observations in our study highlighted that patients from the statin-free group were more likely to develop GSD. The few patients from the statin (+) group who eventually developed GSs received lower doses of statins, however. The relationship between statins and gut dysbiosis has been analyzed by many researchers and could be seen as a two-way condition. There are studies which argue that some particularities of the gut microbiota may modulate the human response to statins, and others that associate the use of statins with some alterations of the bacterial populations of the gut microbiome [38,39]. It is possible that statins have particular effects on different bacteria from the gut microbiota and, by consequence, that they do not influence the same specific microbiota-derived substances. Thus, atorvastatin may cause more significant changes in *Lactobacillus*, *Eubacterium*, *Faecalibacterium*, and *Bifidobacterium* and less significant changes in the *Clostridium* genus. Simvastatin may have more influence on the *Bacteroides* phylum and *Lactobacillus* genus and less on the *Firmicutes* phylum. Rosuvastatin does not seem to alter the *Firmicutes/Bacteroidetes* ratio and seems to have an insignificant influence on *Fusobacterium* and *Proteobacteria*, as well as *Ruminococcus* spp., but it has a potent role in favoring *Bacteroides* spp. and *Lachnospinaceae* spp. This particular behavior may partly explain why there are differences related to the efficacy of statin treatment in lowering the lipid level [40]. The results of our study revealed that patients from the group treated with statins displayed a lower range of dysbiosis and a lower risk of GSD. The fact that each statin formulation might act differently at the microbiome level should be taken into consideration. Our study included patients who were treated with different types of statins, with the condition that their use should be longer than five years. Patients diagnosed with GSD, especially those from the nontreated group, exhibited important alterations in the gut microbiota and a higher severity of gut microbiota dysbiosis.

It is already known that dysbiosis of the gut microbiota is a cofactor in the development of overweight, obesity, and many related metabolic conditions, including diabetes and dyslipidemia [41]. For this reason, some studies aimed to demonstrate that a potential therapeutical approach to the metabolic issues could involve addressing the gut microbiota by restoring the microbiome imbalance. Thus, the use of cholesterol-lowering drugs, such as atorvastatin and rosuvastatin, in an animal model demonstrated a significant alteration of some genera such as *Bacteroides*, *Butyricimonas*, and *Mucispirillum*, which corelated well with the inflammatory biomarkers. Moreover, oral fecal microbiota transplantation from the statin-treated mouse groups improved the carbohydrate metabolism. Other studies that addressed the relationship between obesity and gut microbiota alterations rated statin therapy as an important covariate of the microbiome’s diversity and also emphasized that obesity-associated gut microbiota dysbiosis negatively correlated with statin treatment [42]. Our observations revealed that the patients from the statin (+) group displayed significantly lower microbiota dysbiosis. In contrast, the patients from the statin-free group exhibited important alterations in the enterotypes and several bioindicators, with a significant decrease in biodiversity, as well as modifications of the amount of some functional gut microbiota and disturbances of the bacterial metabolism. We noted that patients in the statin-free group displayed a significantly higher range of LPS (+) bacteria, as well as a notable decrease in the amount of mucosa-protective and mucin-degrading bacteria in the microbiota. The bacterial metabolism exhibited important alterations in patients from the statin-free group, and the number of butyrate-producing microbiota was significantly reduced. We observed that some species were significantly modified in patients in the statin-free group. Thus, in the statin-free group, the LPS (+) bacteria *Enterobacter* spp. were found to be significantly elevated, but other functional bacteria, such as *Akkermansia muciniphila*, *Faecalibacterium Prausnitzii*, and *Roseburia* spp., were found to have significantly decreased ranges.

As some studies have hypothesized, it is also possible that the anti-inflammatory capabilities of statins are positively influenced by some gut microbiota species [43]. Some epidemiologic data reported that pro-inflammatory cytokines, represented by interleukins (IL) such as IL-6, IL-10, IL-12, and IL-13, are associated with a high risk of ischemic heart disease, as well as of GSD [44]. Our results highlighted that the patients from the statin (+) group exhibited a significantly lower range of inflammation biomarkers. From the perspective of the present study, we observed that only a few patients from the statin (+) group underwent cholecystectomies, with most of them having either silent GSs or mitigated forms of GSD, with no signs of inflammation of the GB in the patient or any indication of urgent surgical intervention.

Over the past decades, many studies have emphasized the association between dysbiosis and various digestive conditions, either functional or organic [45,46,47,48,49,50,51,52]. GSD is one of many digestive diseases that are frequently associated with gut microbiota dysbiosis, either in humans or in experimental animal models. The presence of various alterations in the gut microbiota’s characteristics might be an epiphenomenon or could actually play a pathogenetic role in the development of GSs. Some beneficial entities from mucin-degrading bacteria, such as *Akkermansia muciniphila*, were linked in healthy people with a low body weight and a low fat proportion, a low level of adipose tissue inflammation, and reduced insulin resistance. The secretion of intestinal abnormal mucin as a consequence of the malfunctioning of the functional mucin-degrading bacteria may represent risk factors for GSD. Recent studies reported that particular forms of mucins such as mucin-4 may be involved in GSD [53,54]. The lack of *Akkermansia muciniphila* seems to be associated with metabolic issues such as diabetes, obesity, and atherosclerosis with aortic lesions. In contrast, the abundance of *Akkermansia muciniphila* in the gut microbiota may improve the metabolic condition of patients, reducing secondary inflammation to endotoxemia, decreasing the adiposity and insulin resistance, and ensuring a good glucose tolerance. In this case, the addition of this useful bacterium to new probiotic formulations, such as a next-generation probiotic, may represent a promising future treatment alternate [55,56].

Various alterations in the gut microbiome have been reported by different authors to be associated with GSD. Some authors found that *Desulfovibrionals* spp. were significantly increased in patients with GSD. Moreover, it seems that fecal transplantation from patients affected by GSD to a gallstone-resistant strain of mice may promote GS formation. It is possible that *Desulfovibrionales* spp. favors the production of cecal secondary BAs, resulting in higher intestinal cholesterol absorption. Many researchers reported that in populations with GSD, the diversity of the gut microbiota was often severely decreased, as were some gut bacteria, especially those from the *Firmicutes* phylum. These aspects were also associated with an important increase in the secondary BAs from the enterohepatic cycle. The authors suggested that some bacteria, such as *Ruminococcus gnavus* spp., could even be used as biomarkers to point to the group that is exposed to GS formation [57,58,59,60,61,62].

The results of the present study displayed that the statin (−) group exhibited a significantly higher incidence of gut dysbiosis, with important disturbances of the diversity of the microbiota. Patients from the statin-free group exhibited a significant decrease in the Shannon–Weiner H index of their alpha biodiversity. The most observed enterotype in the dyslipidemic population, who did not receive statin therapy, was enterotype I, where the *Bacteorides* spp. were dominant, whereas *Prevotella* spp. and *Ruminococcus* spp. were markedly diminished. An important imbalance of various functional and metabolic microbiota was also noted in the statin-free group, which is associated with a higher incidence of GSD.

The fact that the multivariate analysis showed that DB was a significant independent risk factor for GSD in patients treated with statins emphasized once again that alterations of the gut microbiota play an important role in the development of GSs and must be addressed, even in patients that already have their cholesterol metabolism under control. Some authors hypothesized that GSD might be an expression of systemic imbalances. These multiple systemic pathways may link GSD to various conditions associated with metabolic syndrome, such as insulin resistance, obesity, type 2 diabetes, and nonalcoholic fatty liver disease [63,64]. Our study showed that the long-term use of statins was associated with a lower frequency of GSD. Even though patients from the treated group exhibited a lower range of DB, alterations in the gut microbiota seemed to be an independent risk factor for GSD, along with obesity. These risk factors should be actively addressed through lifestyle changes, dietary plans, and customized pre-/probiotic supplements. 

A full understanding of the pathogenesis of cholesterol-rich GSs could possibly reshape not only the management of this condition but also its prevention [65,66,67].

### Limitations

The present study has limitations, however. Some of them are due to the relatively small number of research participants and others because of the cross-sectional study design, with all the biases and drawbacks that originate from that. Possible confounders could also have intervened given the large number of patients with metabolic issues, which are known for their frequent associations with gut microbiota alterations. Other biases could also have emerged from the difficulties of assessing the exact level of the lifestyle changes made by these dyslipidemic patients, whether treated with statins or not, as well as their consequences in terms of modifications of the gut microbiota. The dietary changes made by the compliant patients, characterized by a reduction in fat and increase in fiber intake, might have an impact on the gut microbiota’s footprint, which could be difficult to quantify. It should also be taken into account that each statin formulation might alter the gut microbiota in a different way, and the present study enrolled patients who were treated with various kinds of statins at different dosages. We should also mention that we did not use metagenomic methods, but cultural ones, which may have errors associated with different survival rates of the intestinal bacteria under aerobic conditions. Therefore, these results should be carefully interpreted, while causality cannot be demonstrated based on an observational study. However, it is quite possible that large prospective studies can validate the present results in the future.

## 5. Conclusions

The long-term use of statins in dyslipidemic patients was associated with a low risk of developing GSD. The gut microbiota in dyslipidemic patients following the long-term use of statins was characterized by a low risk of developing an imbalance of various functional bacteria and of alterations in the metabolic microbiota. DB and obesity were found to be independent risk factors predicting GSD in statin (+) patients.

## Figures and Tables

**Table 1:** Demographic baseline data of research participants.

Demographic Data	Statin (+)	Statins (−)	CON	p_1_	p_2_	p_3_
Age	60.92 ± 14.85	58.36 ± 11.76	57.77 ± 10.52	0.1781	0.0850	0.7089
Gender F/M	52/48	51/49	54/46	0.8878	0.7774	0.6718
Urban residency	70%	62%	71%	0.2336	0.8771	0.1786
Working/retired	45%55/%	51%/49%	52/48%	0.3969	0.3232	0.8878

Notes: CON = controls, F = females, M = males, p_1_ = statin (+) vs. statin (−), p_2_ = statin (+) vs. CON, p_3_ = statin (−) vs. CON.

**Table 2:** Biological workups of research participants.

Biological Workups	Statin (+)	Statins (−)		p_1_	p_2_	p_3_
Hemoglobin (g/dL)	13.306 ± 1.395	13.279 ± 0.745	13.433 ± 1.451	0.1538	0.6128	0.2081
Leukocytes/mm^3^	8.775 × 10^3^ ± 3.017 × 10^3^	8.750 × 10^3^ ± 2.985 × 10^3^	7.983 × 10^3^ ± 2.627 × 10^3^	0.7275	0.0712	0.0660
Platelets/mm^3^	264.71 × 10^3^ ± 48.37 × 10^3^	256.06 × 10^3^ ± 69.79 × 10^3^	257.35 × 10^3^ ± 32.72 × 10^3^	0.3096	0.2090	0.8660
CRP (U/L)	0.86 ± 0.57	0.94 ± 0.30	0.65 ± 0.43	0.2157	0.0037	<0.0001
ALT (IU/L)	21.92 ± 5.14	22.43 ± 4.09	21.62 ± 4.17	0.4384	0.6509	0.1617
Conjugated Bilirubin (mg/dL)	0.36 ± 0.26	0.39 ± 0.25	0.37 ± 0.12	0.4066	0.7273	0.4716
Amylase (U/L)	20.43 ± 5.96	20.03 ± 5.26	19.56 ± 3.46	0.6154	0.2083	0.4562
FPG (mg/dL)	102.36 ± 9.57	99.03 ± 7.45	92.36 ± 4.57	0.0066 *	<0.0001 *	<0.0001 *
Creatinine (mg/dL)	0.706 ± 0.254	0.703 ± 0.145	0.69 ± 0.211	0.0449	0.7597	0.6924
LDL cholesterol	100.22 ± 2.81	172.81 ± 12.64	95.54 ± 2.81	<0.0001 *	<0.0001 *	<0.0001 *
Triglycerides	109.76 ± 2.31	114.96 ± 3.01	101.42 ± 2.11	<0.0001 *	<0.0001 *	<0.0001 *
Gut dysbiosis	35%	54%	3%	0.0070 *	<0.0001 *	<0.0001 *

Notes: CON = controls, CRP = C-reactive protein, ALT = alanine aminotransferase, FPG = fast plasma glucose, LDLs = low-density lipoproteins, g/dL = grams/deciliter, mm^3^ = millimeter^3^, U/L = units/liter, IU/L = international units/liter, mg/dL = milligrams/deciliter, p_1_ = statin (+) vs. statin (−), p_2_ = statin (+) vs. CON, p_3_ = statin (−) vs. CON, * = statistically significant.

**Table 3:** Clinical data on dyslipidemic patients.

Clinical Spectra	Statin (+)	Statins (−)	* p *
Admission by ER	41%	45%	0.5688
Smoking history	48%	45%	0.6714
Alcohol consumption history	39%	28%	0.1002
Dyslipidemia duration (years)	14.25 ± 1.3	13.25 ± 1.2	<0.0001 *
Family history of GSD	23%	35%	0.0621
Female postmenopausal estrogen replacement therapy	17%	11%	0.2226
DM/IGT	35%	29%	0.3643
Metformin treatment	9%	4%	0.1526
Obesity	45%	38%	0.3163
Hypertension	22%	17%	0.3734
Other CV conditions	30%	21%	0.1453
IBS	2%	4%	0.4083
NAFLD	42%	36%	0.3856
GSD	5%	14%	0.0304 *
Cholecystectomy	1%	3%	0.3136
Good in-hospital outcome	85%	80%	0.3533

Notes: ER = emergency room, GSD = gallstone disease, DM = diabetes mellitus, IGT = impaired glucose tolerance, CV = cardiovascular, IBS = irritable bowel syndrome, NAFLD = nonalcoholic fatty liver disease, GSD = gallstone disease, * = statistically significant.

**Table 4:** Alterations to stool microbiota.

Alterations to Microbiota in Dysbiotic Patients	Statin (+)	Statin (−)	* p *
Dysbiosis severity	1.42 ± 0.60	2.05 ± 0.65	<0.0001 *
Biodiversity Shannon–Wiener H index	2.68 ± 0.51	2.21 ± 0.23	<0.0001 *
Enterotype 1	14/35 (40%)	36/54 (66.66%)	0.013 *
Enterotype 2	13/35 (37.14%)	12/54 (22.22%)	0.1282
Enterotype 3	7/35 (20%)	4/54 (7.4%)	0.0793
Enterotype unclassified	1/35 (2.85%)	2/54 (3.7%)	0.8290
Increased LPS (+) bacteria	21/35 (60%)	48/54 (88.88%)	0.0148 *
Decreased mucin-degrading microbiota	22/35 (62.85%)	45/54 (83.33%)	0.0296 *
Decreased mucosa-protective microbiota	23/35 (65.71%)	47/54 (87.03%)	0.0171 *
Decreased butyrate-producing microbiota	24/35 (68.57%)	47/54 (87.03%)	0.0352 *

Notes: LPS = lipopolysaccharide, * = statistically significant.

**Table 5:** Clinical data on patients with GSD.

Age		59.40 ± 2.30	59.43 ± 1.74	0.9772
Females		4	9	0.5275
Males		1	5	0.5278
Symptoms and signs	asymptomatic	3	5	0.3580
	dyspepsia	1	5	0.5278
	jaundice	0	1	0.5508
	Murphy’s sign	1	3	0.9481
US GS features	<1 cm	3	2	0.05 *
	1–3 cm	1	11	0.0233 *
	>3 cm	0	1	0.5508
	multiple	4	11	0.9478
	solitary	1	3	0.9481
Cholecystectomy		1	2	0.7694
GS composition	cholesterol-rich	2	5	0.1224

Notes: GSD = gallstone disease, Nb = number, US = ultrasound, GSs = gallstones, * = statistically significant.

**Table 6:** FTIR band assignments.

Code	ν_O-H_	ν_OH/NH_ *	ν^as^_CH2/CH3_	ν^s^_CH2_	ν^s^_CH2/CH3_	ν_C=O_ * _(carboxyl)_	ν_C=C_	ν_C=O_ * _(lactam)_
Cholesterol, CH	3429.1 (±0.9)	-	2931.1 (±0.6)	2900.7 (±0.1)	2866.8 (±0.2)	-	1671.1 (±0.3)	-
Gallstone GS1	3435.9 (±3.5)	3272.9 (±0.2)	2933.4 (±0.2)	2897 (±0.2)	2864 (±0.2)	1699.2 (±0.7)	-	1651.5 (±0.3)
Gallstone GS2	3430.1 (±0.6)	3276 (±1.5)	2933 (±0.3)	2898.2 (±1.3)	2865 (±0.3)	1699.7 (±0.8)	1662.3 (±0.8)	1649.3 (±3.5)
Gallstone GS3a	3395.4 (±1.3)	-	2931.8 (±0)	2899.7 (±0.2)	2866.3 (±0.1)	1699.7 (±1.7)	1667.1 (±0.6)	1649.5 (±2.2)
Gallstone GS3b	3396.1 (±0.9)	-	2931.1 (±0.3)	2900.7 (±0.1)	2866.6 (±0.2)	1699.8 (±0.3)	1667.8 (±0.8)	-
Code	ν_C=C_ *	ν^s^_CH2/CH3_	δ1_CH2/CH3_	δ2_CH2/CH3_	δ_CH2_	δ_wCH2_	δ1_CC/CN_ *	δ_CH2_
Cholesterol, CH	-	1463.7 (±0.1)	1437.8 (±1.8)	1372.8 (±3.4)	1334.1 (±0.8)	1315.5 (±1)	-	1273.5 (±0.6)
Gallstone GS1	1572.4 (±0)	1461.4 (±0.2)	1444.1 (±0.2)	1368.4 (±0.1)	1330.1 (±0.1)	-	1281.4 (±0.2)	-
Gallstone GS2	1572.5 (±0.7)	1462.4 (±0.3)	1442.7 (±1.6)	1368.5 (±1.8)	1332.3 (±0.7)	-	1280.9 (±2)	-
Gallstone GS3a	1574.1 (±0.2)	1463.4 (±0.1)	1440.1 (±0.2)	1371 (±0.3)	1335.1 (±0.4)	-	-	1274.1 (±0.7)
Gallstone GS3b	1571.1 (±0.3)	1463.8 (±0.2)	1438 (±0.9)	1375.6 (±3.5)	1334.9 (±1)	-	-	1272.3 (±0.5)
Code	δ_CC/CN_ *	δ_CH2_	ν_C-H_	δ_C-H_	δ_C-H_	δ_C-H_	δ_Rg_	δ_C-H_
Cholesterol, CH	-	1236.2 (±0.7)	1191 (±0.2)	1168.1 (±1.4)	1131.8 (±0.9)	1107.3 (±0.1)	1054.2 (±0.1)	1022.7 (±0.1)
Gallstone GS1	1250.1 (±0.1)	1235.1 (±0.2)	1196.6 (±0.1)	1169.6 (±0.2)	1135.8 (±0.1)	1109.4 (±0.1)	1048 (±0.1)	1020.2 (±0.1)
Gallstone GS2	1250.7 (±0.8)	1235.8 (±0.9)	1195.5 (±0.2)	1169.3 (±0.8)	1135.1 (±0)	1109.1 (±0.3)	1050 (±0.7)	1021.5 (±0.8)
Gallstone GS3a	1250.4 (±1)	1236.8 (±0.2)	1192.3 (±0.2)	1167.1 (±0.7)	1133.3 (±0.1)	1107.7 (±0.1)	1053.2 (±0)	1022.2 (±0)
Gallstone GS3b	1252.5 (±0.6)	1236.7 (±0.4)	1191.1 (±0.2)	1169.2 (±1.4)	1130.5 (±0.4)	1107.2 (±0.1)	1054.5 (±0.2)	1022.7 (±0.2)
Code	δ_=CH_	δ_wCH=CH2/_ _CH2_	δ_=CH_	δ_CH_	ν_CCC_	δ_CH_	δ_CH_	δ_CH_
Cholesterol, CH	985.9 (±0)	955 (±1.3)	927.5 (±1)	883.4 (±0.6)	839.4 (±0.6)	800.1 (±0.5)	739 (±1.5)	699.5 (±0.5)
Gallstone GS1	983.7 (±0)	954.4 (±0.1)	929.6 (±0.1)	883.4 (±0)	839.6 (±0.1)	798.6 (±0.1)	736.3 (±0.1)	700 (±0.8)
Gallstone GS2	984.9 (±0.6)	954.6 (±0.4)	928.8 (±2.2)	883.4 (±0.1)	839.3 (±0.1)	798.6 (±0.9)	737.1 (±0.6)	699.9 (±0.3)
Gallstone GS3a	985.5 (±0.1)	955.5 (±0.1)	929.7 (±0.4)	882 (±0.3)	839.2 (±0.2)	800.1 (±0)	737.6 (±0.1)	698.5 (±0.5)
Gallstone GS3b	986.3 (±0.4)	955.7 (±0.2)	927.6 (±1.4)	883.1 (±0.3)	839.7 (±0.5)	800.1 (±0.3)	738.6 (±0.8)	699.6 (±0)

Cholesterol band assignments: ν_O-H_, O-H stretching vibrations (~3400 cm^−^^1^); ν^as^_CH2/CH3_, CH_2_ and CH_3_ asymmetric stretching vibrations (~2932 cm^−^^1^); ν^s^_CH2_, CH_2_ symmetric stretching vibrations (2901 cm^−^^1^); ν^s^_CH2/CH3_, CH_2_ and CH_3_ symmetric stretching vibrations (~2860 cm^−^^1^); ν_C=C_, C=C stretching vibrations (~1674 cm^−^^1^); ν^s^_CH2/CH3_, CH_2_ and CH_3_ symmetric stretching vibrations (~1464 cm^−^^1^); δ_CH2/CH3_, CH_2_ and CH_3_ deformation vibrations (~1438 cm^−^^1^); δ_CH2/CH3_, CH_2_ and CH_3_ bending vibrations (~1378 cm^−^^1^); δ_CH2_, CH_2_ bending vibrations (~1331 cm^−^^1^); δ_wCH2_, CH_2_ wagging vibrations (~1317 cm^−^^1^); δ_CH2_, CH_2_ bending vibrations (~1272 cm^−^^1^); δ_CH2_, CH_2_ deformation vibrations (~1236 cm^−^^1^); ν_C-H_, C-C stretching vibrations (~1191 cm^−^^1^); δ_C-H_, in-plane C-H bending vibrations (~1170 cm^−^^1^); δ_C-H_, in-plane C-H bending vibrations (~1131 cm^−^^1^); δ_C-H_, in-plane C-H bending vibrations (~1108 cm^−^^1^); δ_Rg_, ring deformation vibrations (~1055 cm^−^^1^); δ_C-H_, in-plane C-H bending vibrations (~1022 cm^−^^1^); δ_=CH_, =C-H bending vibrations (~985 cm^−^^1^); δ_wCH=CH2/CH2_, CH=CH_2_ and CH_2_ wagging vibrations (~956 cm^−^^1^); δ_=CH_, =C-H bending vibrations (~927 cm^−^^1^); δ_CH_, C-H out-of-plane bending vibrations (~885 cm^−^^1^); ν_CCC_, C-C-C stretching vibrations (~840 cm^−^^1^); δ_CH_, C-H out-of-plane bending vibrations (~800); δ_CH_, C-H out-of-plane bending vibrations (~739 cm^−^^1^); δ_CH_, C-H out-of-plane bending vibrations (~700 cm^−^^1^); δ_=CH_, =C-H out-of-plane bending vibrations (~674 cm^−^^1^). Bilirubin band assignments (* specific bands for bilirubin): ν_OH/NH_, O-H and N-H stretching vibrations (~3398 cm^−1^) or ν_OH/NH_, O-H and N-H stretching vibrations (~3261 cm^−1^); ν^as^_CH2/CH3_, CH_2_ and CH_3_ asymmetric stretching vibrations (~2913 cm^−1^); ν_C=O (carboxyl)_, C=O (from carboxylic group) stretching vibrations (intense) (~1696 cm^−1^); ν_C=O (lactam)_, C=O (from lactam group) stretching vibrations (sharp) (~1647 cm^−1^); ν_C=C_, C=C stretching vibrations (~1575 cm^−1^); δ_CC/CN_, C-C and C-N bending vibrations (~1282 and 1249 cm^−1^); unassigned: 1080 and 1020 cm^−1^; δ_CH3_, CH_3_ bending vibrations (~989 cm^−1^); δ_CH_, C-H bending vibrations (~945 cm^−1^); unassigned: 882 and 630 cm^−1^.

**Table 7:** Multivariate analysis of risk factors for GSD in statin-treated patients.

Table Analyzed	GSD_ Multiple Linear Regression							
Dependent variable	GSD							
Regression type	Least-squares							
Model								
Analysis of Variance	SS	DF	MS	F (DFn DFd)		*p* value		
Regression	8.428	7	1.204	F (7 92) = 15.91		*p* < 0.0001		
DZ/IGT	0.2563	1	0.2563	F (1 92) = 3.386		*p* = 0.0690		
Obesity	0.3728	1	0.3728	F (1 92) = 4.926		*p* = 0.0289		
Alcohol	0.2106	1	0.2106	F (1 92) = 2.783		*p* = 0.0986		
Smoking	0.257	1	0.257	F (1 92) = 3.397		*p* = 0.0685		
Hypertension	0.2352	1	0.2352	F (1 92) = 3.109		*p* = 0.0812		
NAFLD	0.1022	1	0.1022	F (1 92) = 1.351		*p* = 0.2482		
DB	3.707	1	3.707	F (1 92) = 48.98		*p* < 0.0001		
Residual	6.962	92	0.07567					
Total	15.39	99						
Parameter estimates	Variable	Estimate	Standard error	95% CI (asymptotic)	|t|	*p* value	*p* value summary	
β0	Intercept	0.02957	0.05979	−0.08918 to 0.1483	0.4945	0.6221	ns	
β1	DZ/IGT	0.1198	0.06513	−0.009499 to 0.2492	1.84	0.069	ns	
β2	Obesity	0.1361	0.0613	0.01431 to 0.2578	2.22	0.0289	*	
β3	Alcohol	−0.1041	0.0624	−0.2280 to 0.01983	1.668	0.0986	ns	
β4	Smoking	−0.1066	0.05785	−0.2215 to 0.008275	1.843	0.0685	ns	
β5	Hypertension	−0.1124	0.06375	−0.2390 to 0.01421	1.763	0.0812	ns	
β6	NAFLD	0.07831	0.06738	−0.05551 to 0.2121	1.162	0.2482	ns	
β7	DB	0.4417	0.0631	0.3163 to 0.5670	6.999	<0.0001	****	

Notes: * = statistically significant, **** = very highly statistically significant, ns = non-significant.

## Data Availability

The data presented in this study are available on request from the corresponding author.

## Supplementary Materials

The following supporting information can be downloaded at https://www.mdpi.com/article/10.3390/diagnostics14121234/s1: Figures: S1aS1b, S1c, S1d and S1e.
