# Supplementary figures and images for: Cholesterol Gallstones and Long-Term Use of Statins: Is Gut Microbiota Dysbiosis Bridging over Uncertainties?
Source: Diagnostics (Basel). 2024 Jun 12;14(12):1234. doi: 10.3390/diagnostics14121234 (PMC11202934; doi:10.3390/diagnostics14121234)

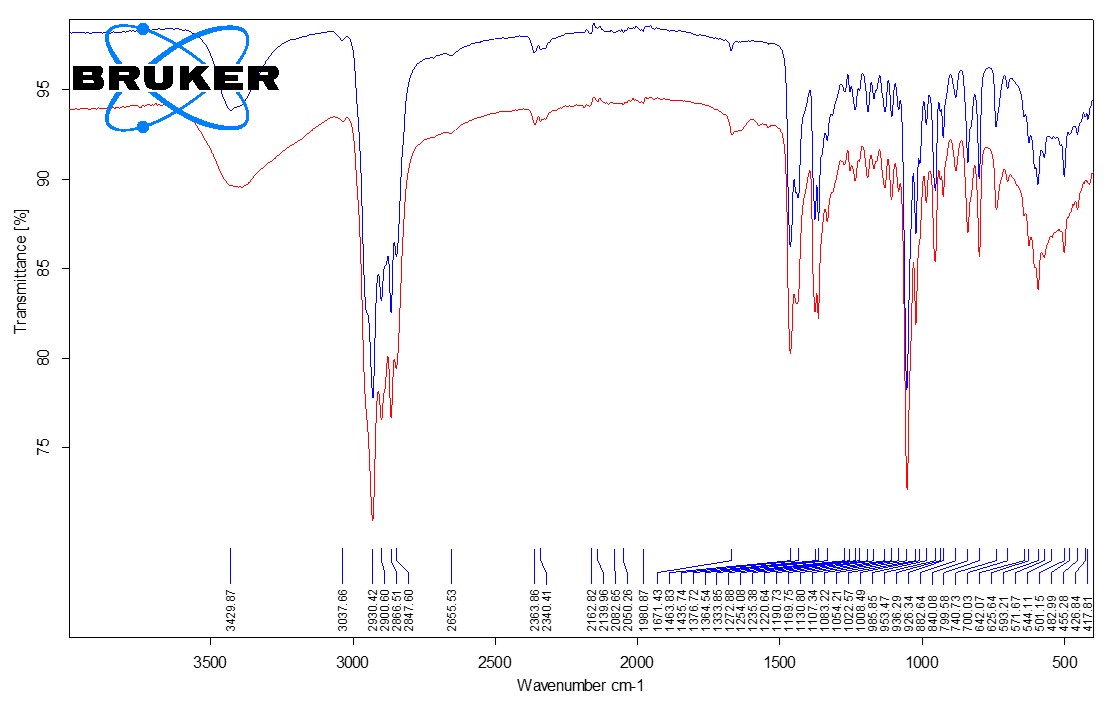

Supplement: Supplementary file 1 [file diagnostics-14-01234-s001.zip › FigureS1d.jpg]

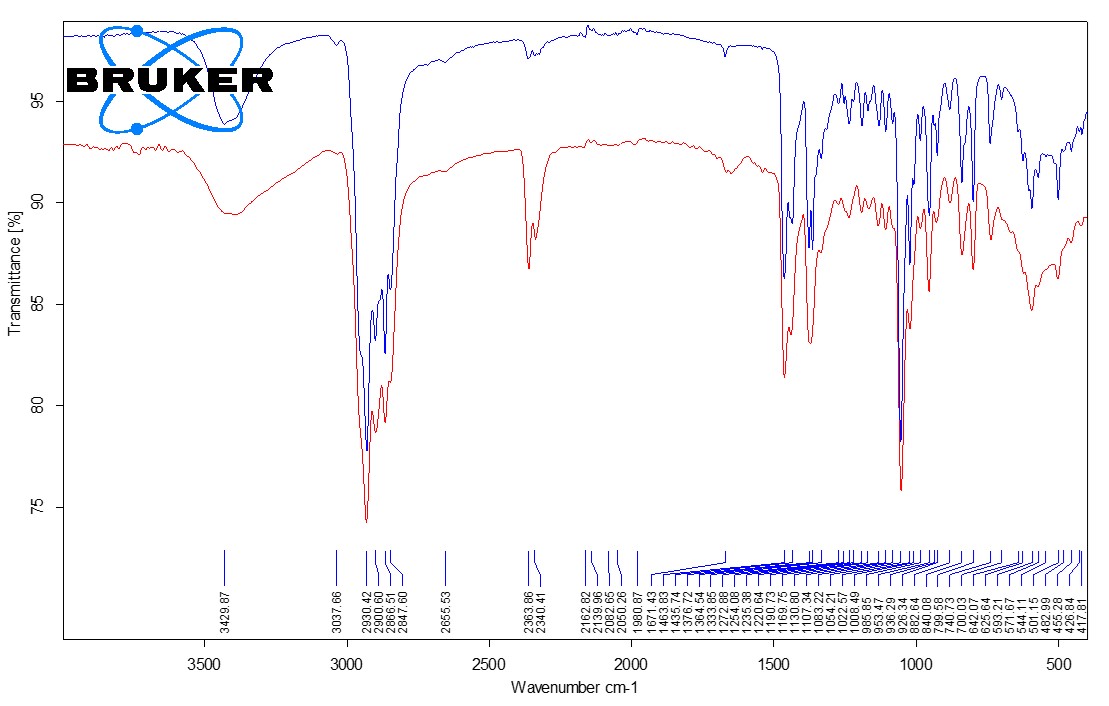

Supplement: Supplementary file 1 [file diagnostics-14-01234-s001.zip › FigureS1e.jpg]

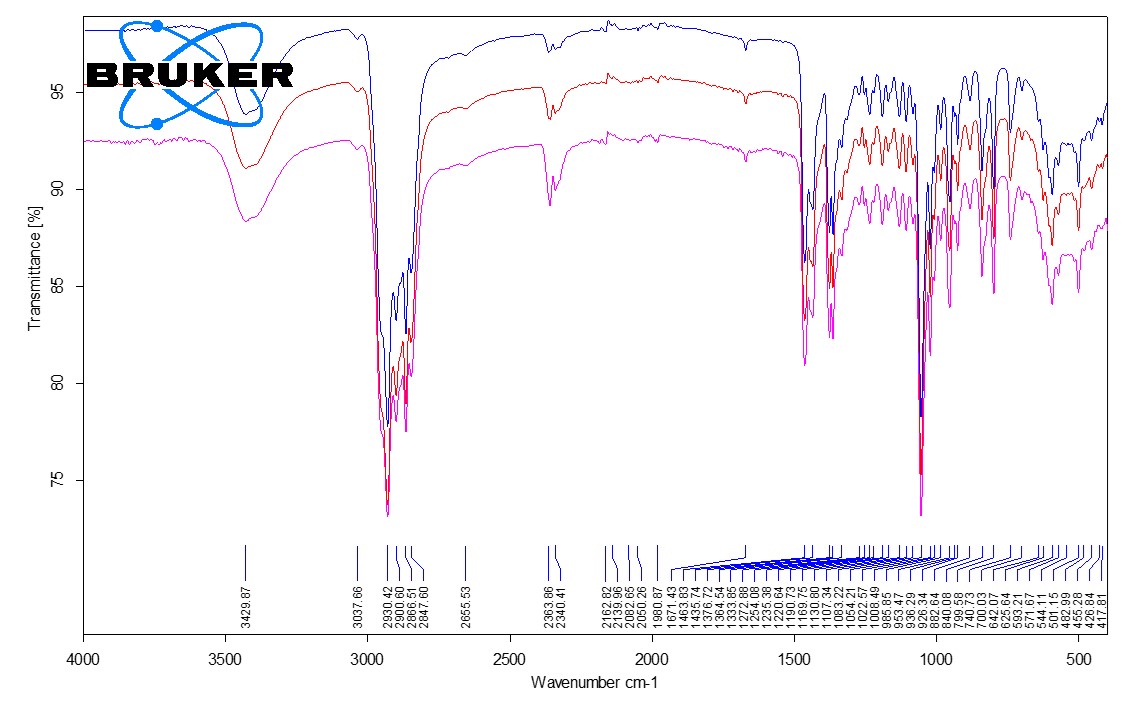

Supplement: Supplementary file 1 [file diagnostics-14-01234-s001.zip › FigureS1a.jpg]

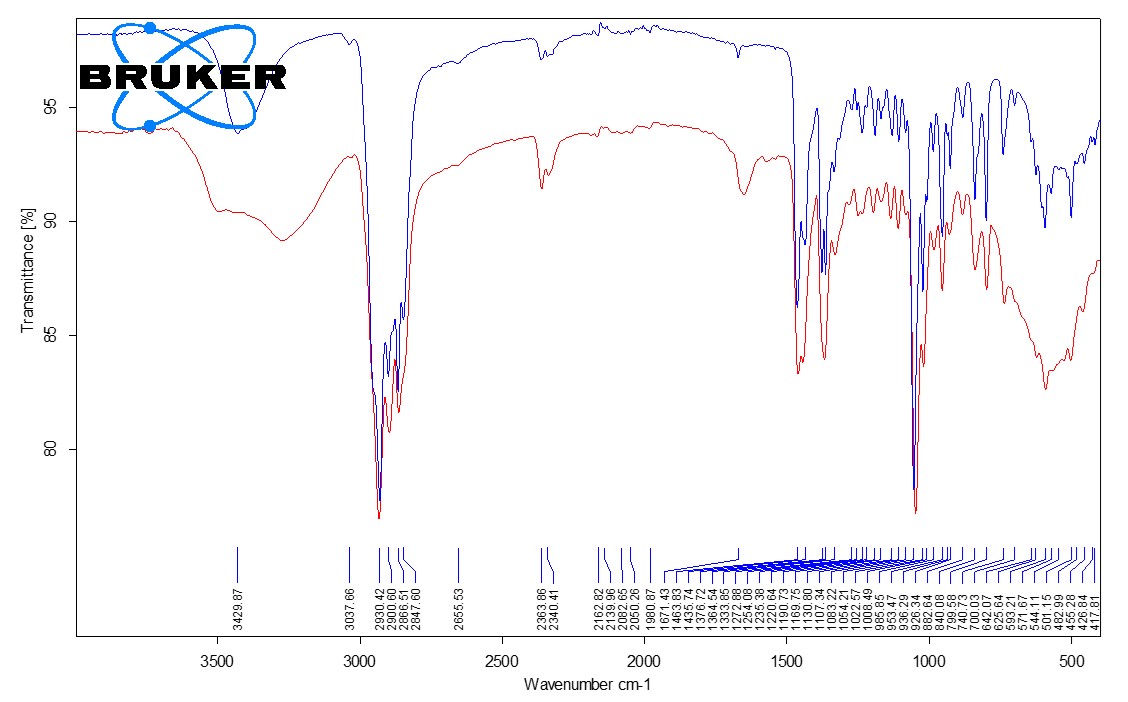

Supplement: Supplementary file 1 [file diagnostics-14-01234-s001.zip › FigureS1b.jpg]

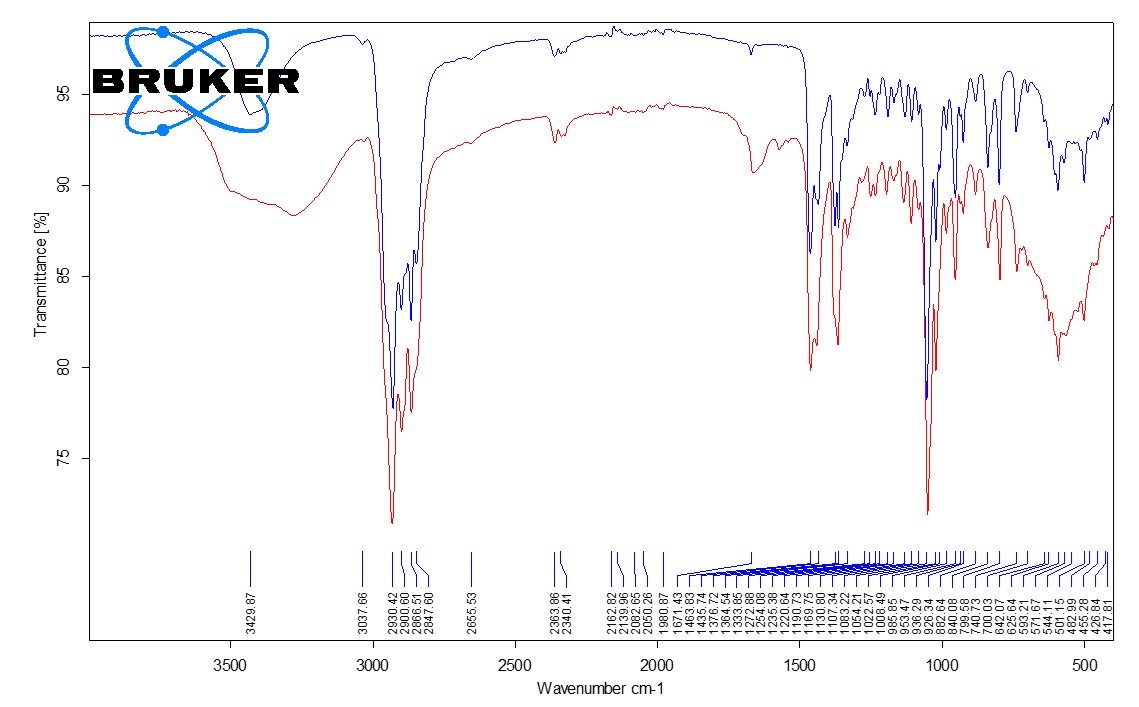

Supplement: Supplementary file 1 [file diagnostics-14-01234-s001.zip › FigureS1c.jpg]
